# Glycogenic Hepatopathy: A Reversible Complication of Uncontrolled Diabetes Mellitus

**DOI:** 10.7759/cureus.9323

**Published:** 2020-07-21

**Authors:** Muhammad N Yousaf, Hamid Ehsan, Sajid Ehsan, Usman Sagheer, Fizah Chaudhary

**Affiliations:** 1 Internal Medicine, MedStar Union Memorial Hospital, Baltimore, USA; 2 Internal Medicine, MedStar Franklin Square Medical Center, Baltimore, USA; 3 Internal Medicine, MedStar Good Samaritan Hospital, Baltimore, USA; 4 Section of Digestive Diseases, Yale School of Medicine, New Haven, USA; 5 Internal Medicine, Anne Arundel Medical Center, Annapolis, USA; 6 Internal Medicine, Medstar Union Memorial Hospital, Baltimore, USA

**Keywords:** glycogenic hepatopathy, type i diabetes mellitus, liver dysfunction, diabetic ketoacidosis, nonalcoholic fatty liver disease, liver biopsy, periodic acid-schiff staining, transaminitis

## Abstract

Glycogenic hepatopathy (GH) is a rare complication of long-standing uncontrolled type I diabetes mellitus (TIDM) resulting in liver dysfunction and hepatomegaly due to intrahepatic deposition of glycogen. Herein we present a 19-year-old male with a history of TIDM and multiple prior hospitalizations with diabetic ketoacidosis (DKA) who presented with nausea, vomiting, right upper quadrant pain, and massive hepatomegaly. Laboratory workup was consistent with DKA and revealed a greater than 10-fold increase in liver enzymes. Despite the resolution of DKA, his liver function was worsening, and further workup was indicated. Ultimately, he underwent a liver biopsy that showed swollen hepatocytes overloaded with intracytoplasmic glycogen consistent with glycogenic hepatopathy. It is an underestimated entity and physicians should have a high index of suspicion for GH in individuals presenting with liver dysfunction, hepatomegaly, and poor glycemic control in TIDM. Strict glycemic control may result in complete resolution of disease.

## Introduction

Glycogenic hepatopathy (GH) is a rare complication of type I diabetes mellitus (TIDM), resulting in abnormal deposition of glycogen in the liver due to poor glycemic control. Also known as Mauriac syndrome, it was first described by Pierre Mauriac in 1930 as a disease of the pediatric population with uncontrolled TIDM and obesity characterized by derangement of liver enzymes, hepatomegaly, cushioned appearance, puberty delay, and growth failure [1]. Over the past decade, the term "glycogenic hepatopathy" has become a universally known entity of hepatocytic accumulation of glycogen, which may be seen in adults without extrahepatic manifestation of Mauriac syndrome [2]. Common predisposing factors of GH are female gender, younger age, TIDM, and rarely insulin-dependent type II diabetes mellitus (TIIDM) [3]. In non-diabetics, GH may be associated with anorexia nervosa, dumping syndrome after gastric bypass, and use of azathioprine or high-dose steroid therapy.

GH is associated with a wide range of clinical manifestations, ranging from asymptomatic liver dysfunction to symptoms associated with elevated blood sugars and diabetic ketoacidosis (DKA) such as polyuria, polydipsia, abdominal pain, nausea, vomiting, acute hepatitis, jaundice, and pruritus. In the younger age group, it presents with significant hepatomegaly, growth failure, and delayed puberty. In extreme cases, with excessive glycogen storage and poor glycemic control over a long period of time, it can cause sinusoidal compression and manifest as ascites [4]. GH is still an underrecognized and commonly missed diagnosis due to a similar presentation to the more common disorder of non-alcoholic fatty liver disease (NAFLD) in patients with poorly controlled diabetes. The radiological differentiation of GH from NAFLD and other glycogen storage diseases is challenging [5]. Liver biopsy is diagnostic, and pathological accumulation of cytoplasmic glycogen is a characteristic feature on periodic acid-Schiff (PAS) staining, which disappears with diastase. GH is a reversible disease with a potential for complete resolution with optimal glycemic control [1]. 

## Case presentation

A 19-year-old male with a history of poorly controlled TIDM, hypertension, and multiple prior hospitalizations with DKA presented to the emergency room with nausea, multiple episodes of non-biliary, non-bloody vomiting, and epigastric and right upper quadrant (RUQ) abdominal pain. Physical examination was remarkable for tachycardia to 110 per minute, abdominal distension, and epigastric and RUQ tenderness with palpable inferior margins of liver indicating hepatomegaly. There was no evidence of ascites, splenomegaly, or tenderness at costophrenic angles. Laboratory workup (Table 1) revealed elevated lactic acid (5.7 mmol/L), anion gap (20 mmol/L), blood sugar (265 mg/dL), and glycated hemoglobin (HbA1c; 14.0%). Urine analysis was positive for ketones. He had abnormal liver function tests with an elevated aspartate transaminase (AST; 247 units/L), alanine aminotransferase (ALT; 264 units/L), alkaline phosphatase (212 units/L), and normal direct bilirubin (0.27 mg/dL). His workup was consistent with DKA due to medication non-adherence. During hospitalization he was managed medically, resulting in complete resolution of DKA, however, his liver function continued to worsen which prompted a further workup. His autoimmune workup was positive for antinuclear antibody (ANA) with a titer of 1:320 but was negative for anti-smooth antibody, antimitochondrial antibody, liver/kidney microsome type 1 antibody, and liver cytosol autoantibodies. Viral serologies were negative for hepatitis B, hepatitis C, human immunodeficiency virus (HIV), Epstein-Barr virus (EBV), influenza A, and influenza B. Workup for hemochromatosis, such as serum ferritin, iron, total iron-binding capacity, and transferrin, was unremarkable; therefore, the genetic test for hemochromatosis (Hemochromatosis (HFE) 3 Mutations) was not pursued. Serum ceruloplasmin, copper concentration, urinary 24-hour copper, and copper to creatinine ratio were normal. Slit-lamp ophthalmic examination for Kayser-Fleischer rings was negative, indicating no evidence of Wilson disease. Toxicology studies for serum acetaminophen, alcohol, and urine drug screen were unremarkable (Table 1).

**Table 1:** Laboratory workup H, high; N, normal; L, low

Laboratory Test	Reference range	Results at presentation
Liver function tests		
Alanine aminotransferase (ALT)	15-41 u/L	264 (H)
Aspartate aminotransferase (AST)	3-34 u/L	247 (H)
Alkaline phosphatase	45-117 u/L	212 (H)
Direct bilirubin	0.2-1.3 mg/dl	0.27 (N)
Total Protein	6.3-8.2 g/dl	7.5 (N)
Albumin	3.5-5.0 g/dl	2.9 (L)
Coagulation Studies		
Prothrombin time (PT)	10-13.5 seconds	13.3 (N)
Partial thromboplastin time	23.4-36.2 seconds	24.6 (N)
International normalized ratio (INR)	0.8-1.2	1.0 (N)
Viral serologies		
Hepatitis A, IgM	Nonreactive	Nonreactive
Hepatitis A, IgG	Nonreactive	Nonreactive
Hepatitis B, core IgM	Nonreactive	Nonreactive
Hepatitis B, surface antigen	Nonreactive	Nonreactive
Hepatitis C antibody	Nonreactive	Nonreactive
HIV 1/0/2 antibody/antigen	Nonreactive	Nonreactive
Epstein-Barr virus	Negative	Negative
Influenza A	Negative	Negative
Influenza B	Negative	Negative
Autoimmune liver disease panel		
Antinuclear antibody	Negative	Positive
Antinuclear antibody titer	<1:40 U	1:320 (H)
Antismooth muscle antibody-Mayo	Negative	Negative
Antismooth muscle antibody IgG-Mayo	<1.0 U	<0.2 U (N)
Antimitochondrial antibody, M2	<0.1 U	<0.1 (N)
Liver/Kidney Microsome Type 1 Ab	<20.0 U	<0.5 U (N)
Liver Cytosol Autoantibodies	<15 U/mL	<5.0 U/ml(N)
Copper level	0.75-1.45mcg/mL	0.83 (N)
Ceruloplasmin	20-60 mg/dL	19 (L)
Urinary copper	0-49 mcg/gm Cr	6 (N)
Urinary copper/creatinine (Cr) ratio	3-35 mcg/24 hr	19 (N)
Urinary 24-hour (hr)copper	31-200 mg/dL	24 (N)
Haptoglobin	49-181 mcg/dL	218 (H)
Iron level	261-462 mcg/dL	239 (L)
Total iron binding capacity	20-55%	33 (N)
Iron concentration	28-365 ng/mL	300 (N)
Ferritin	200-360 mg/dL	211 (N)
Transferrin	0-49 mcg/gm Cr	19 (N)
Toxicology studies		
Acetaminophen level	10-30 mcg/ml	<2 (N)
Ethanol level	0-3 mg/dL	<3 (N)
Urine toxicology screen	Negative	Negative

**Figure 1:**
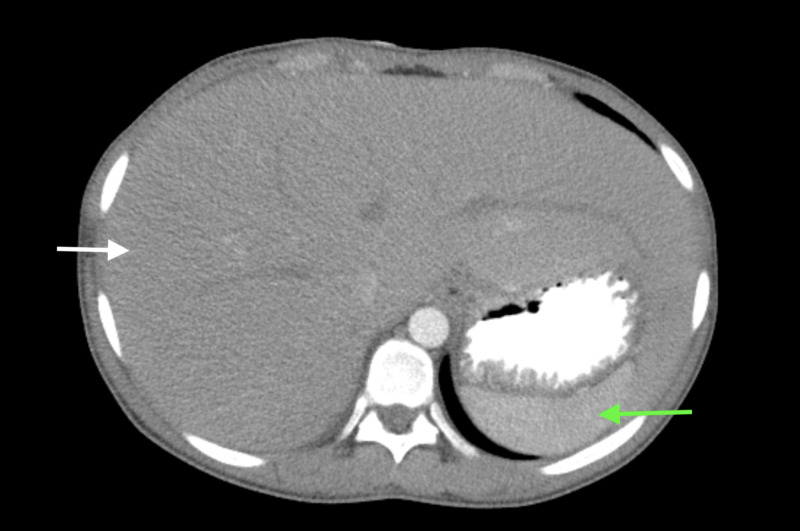
Computed tomography (CT) of the abdomen A transverse view of abdominal CT scan showing massive hepatomegaly (white arrow) with mild diffuse nonspecific hepatic steatosis as indicated with the difference in intensity between liver (white arrow) and spleen (green arrow).

**Figure 2:**
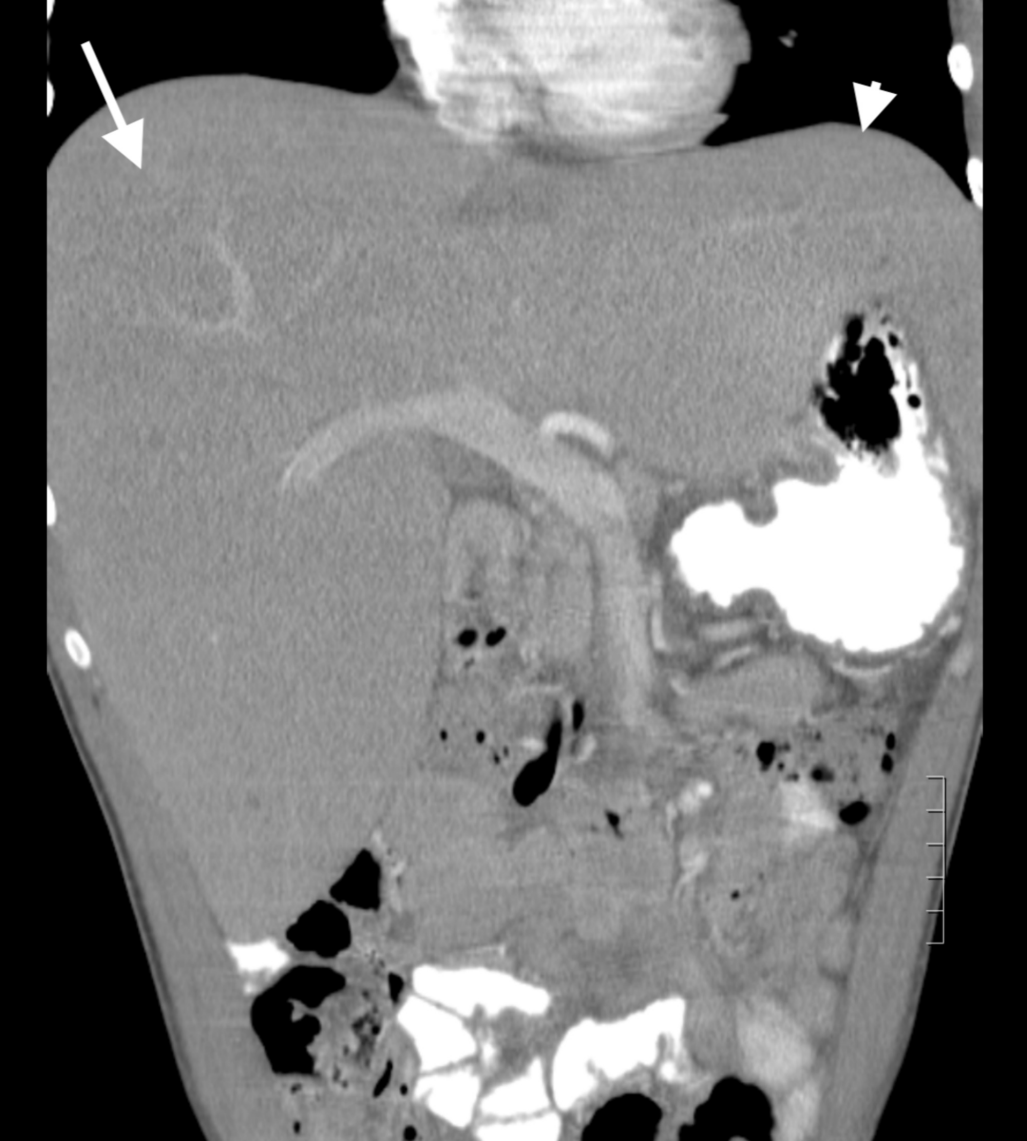
CT scan of the abdomen A coronal section of the abdominal CT scan showing massive hepatomegaly with the right lobe (white arrow) pushing down the bowel and the left lobe of liver extending up to the left upper quadrant of the abdomen (white arrowhead).

Ultrasound of the abdomen with doppler showed an enlarged liver measuring up to 23 cm with increased echotexture reflecting fatty infiltration. Computed tomography (CT) of the abdomen and pelvis with intravenous contrast (Figures 1, 2) showed marked hepatomegaly with diffuse nonspecific hepatic steatosis.

**Figure 3:**
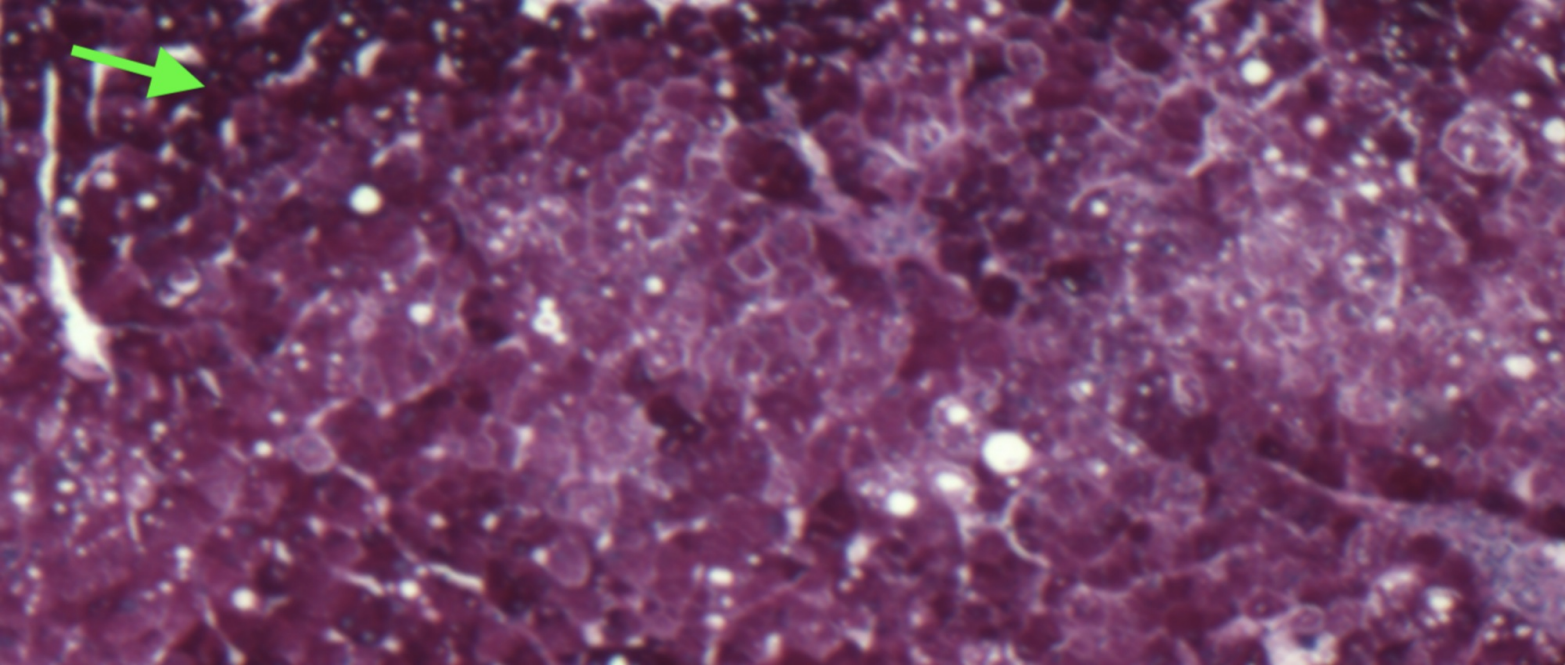
Periodic acid-Schiff (PAS) staining of liver biopsy A strong cytoplasm staining of hepatocytes (arrow) indicating an extensive glycogen deposition.

**Figure 4:**
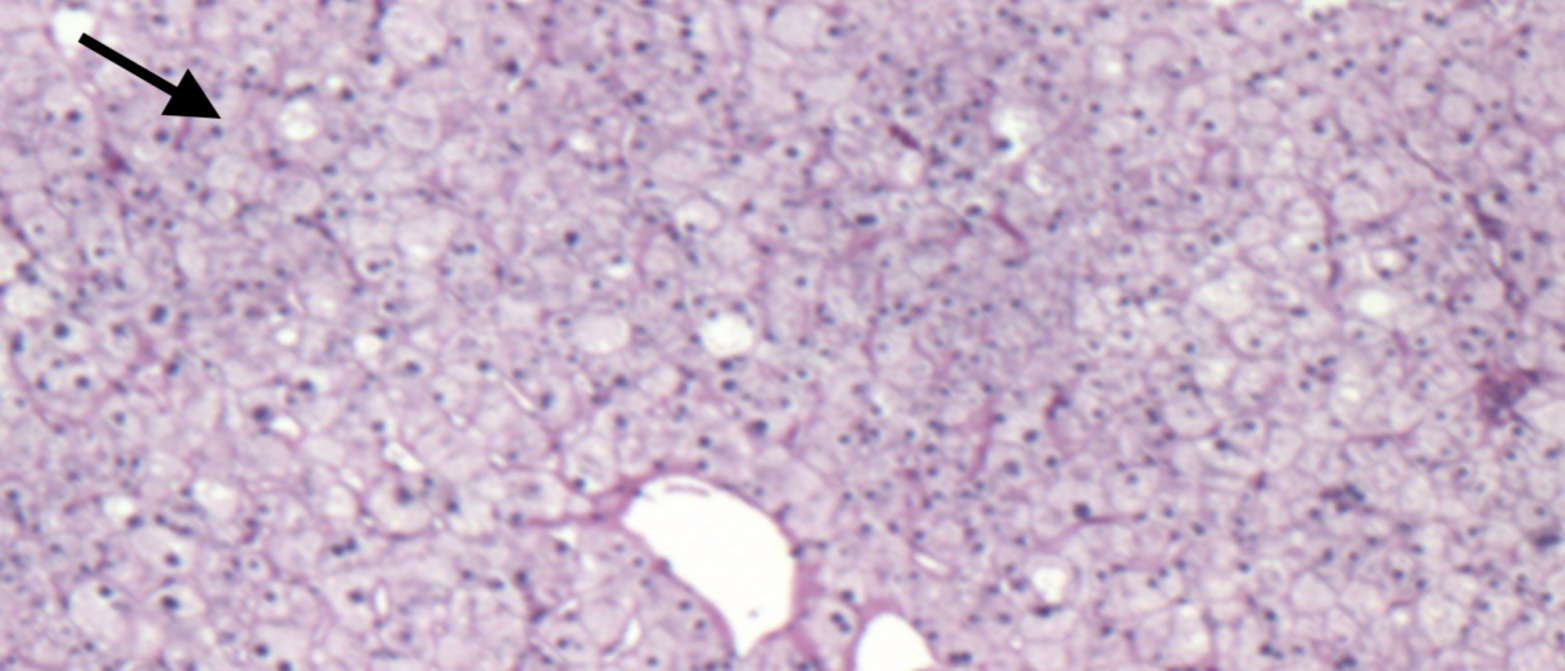
Periodic acid-Schiff diastase (PAS-D) staining Intracytoplasmic periodic acid-Schiff staining disappeared upon treatment with diastase (PAS-D stain), confirming the pathological glycogen accumulation within the hepatocytes.

A liver biopsy demonstrated a marked deposition of hepatocellular glycogen, mild steatosis, and nonspecific chronic inflammation without evidence of significant fibrosis. While iron staining of the biopsy specimen was negative, however, periodic acid-Schiff (PAS) staining showed the presence of abundant cytoplasmic glycogen within the hepatocytes (Figure 3). Washing out of PAS staining with diastase (PAS-D) resulted in the disappearance of intracytoplasmic glycogen due to the enzymatic digestion, confirming the diagnosis of GH (Figure 4).

**Figure 5:**
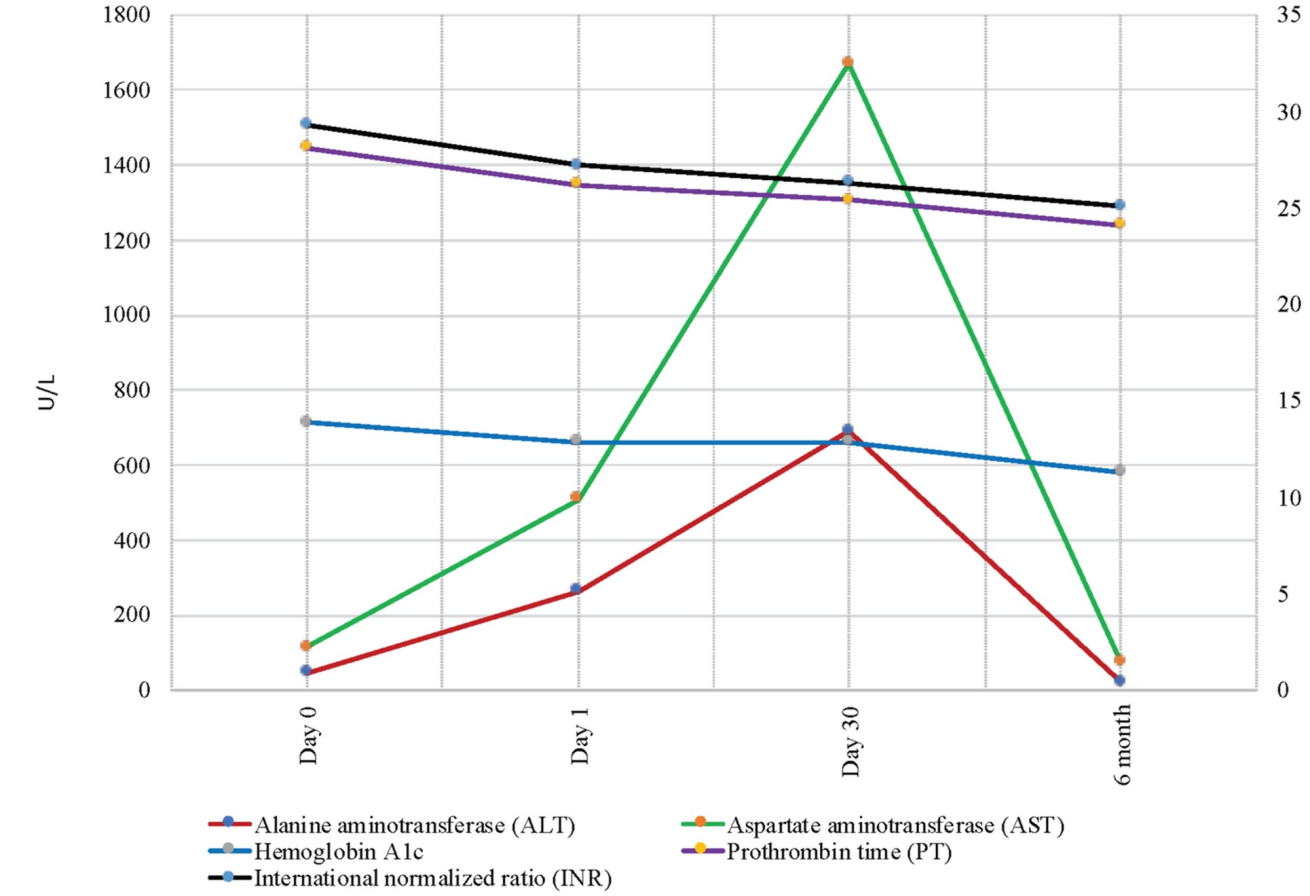
Follow-up laboratory tests Trends of liver function test, hemoglobin A1c, prothrombin, and INR from day 0 to six months.

He was managed with supportive care and strict glycemic control. His clinical symptoms and hepatomegaly completely resolved, and liver biochemistry normalized at six months follow-up (Figure 5).

## Discussion

**Figure 6:**
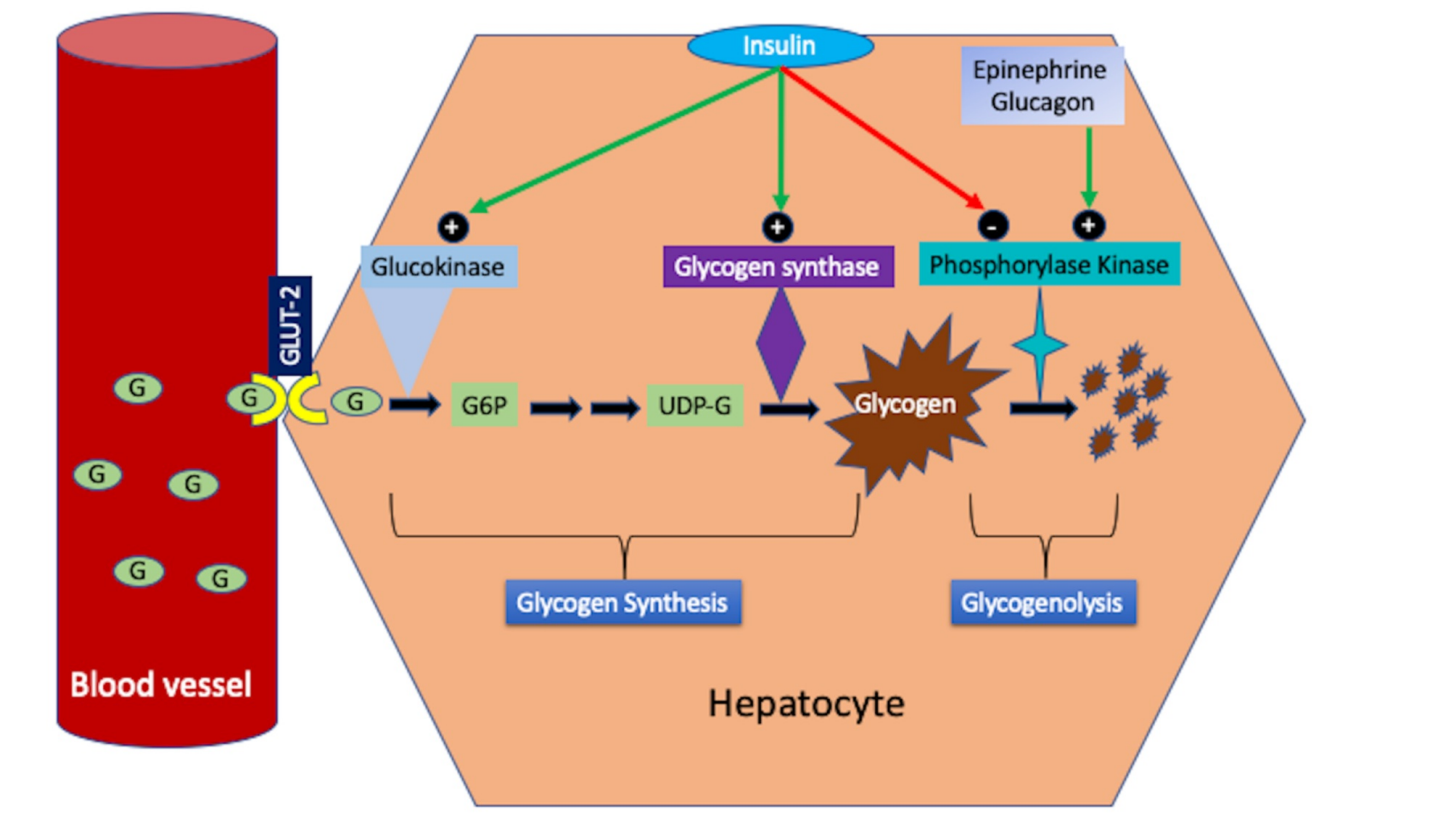
Mechanism of glycogen deposition in hepatocytes under the effects of insulin Blood glucose enters hepatocytes through the GLUT-2 transporter. Insulin activates glucokinase which entraps glucose in the hepatocytes in the form of G6P. Insulin activates glycogen synthase resulting in glycogenesis and indirectly inhibits the effects of epinephrine and glucagon by deactivation of phosphorylase kinase that prevents glycogenolysis. G, Glucose; GLUT-2, glucose transporter 2; G6P, glucose-6-phosphate; UDP-G, uridine diphosphate glucose; +, stimulate; -, inhibit.

GH is a rare but reversible complication of TIDM, and the exact incidence of the disease is unknown. Persistent hyperglycemia and a high dose of insulin are the driving factors of intrahepatic glycogen deposition resulting in hepatomegaly and liver dysfunction. Insulin and hepatic glucose transporter 2 (GLUT-2) are the main regulators of intra- or extrahepatic glucose transport to maintain a glucose equilibrium between serum and hepatocytes. In a hyperglycemic state, glucose may passively diffuse into hepatocytes without insulin or GLUT-2 regulation. In patients with poorly controlled TIDM and TIIDM, a high dose of insulin stimulates hepatic glycogenesis by several mechanisms (Figure 6). First, insulin activates hepatic glucokinase, an enzyme which traps the blood glucose within the hepatocytes by phosphorylation of glucose to glucose-6-phosphate (G6P) [6]. Second, insulin inhibits glycogenolysis by inactivation of hepatic phosphorylase kinase, an enzyme that stimulates glycogen breakdown under the effect of glucagon and epinephrine [6]. Insulin indirectly inhibits the effects of glucagon and epinephrine and hence glycogenolysis. Third, insulin and hyperglycemia stimulate a high concentration of phosphatase, an enzyme that activates hepatic glycogen synthase resulting in the conversion of G6P to glycogen [6].

Common clinical symptoms at presentation are RUQ abdominal pain, nausea, vomiting, and occasionally abdominal distension due to massive hepatomegaly. A large systematic review, including 127 patients, reported RUQ tenderness (46.8%) and hepatomegaly (81.7%) as predominant clinical findings on physical examination [3]. TIDM patients with a high HbA1c and an elevated liver enzyme are suspected of having GH. It should be differentiated from NAFLD and other glycogen storage diseases that may present with similar clinical features. A predominant elevation of hepatocellular liver enzymes with or without cholestatic components and preservation of synthetic function of the liver is the typical reported pattern noted in 93% of patients in a large systematic review [3]. Our patient presented with DKA due to medication non-adherence. Despite the resolution of DKA, his persistent RUQ pain, hepatomegaly, and significantly abnormal liver biochemistry prompted additional testing to rule out other etiologies of liver dysfunction. His ANA was positive; however, his extensive workup for autoimmune diseases, hemochromatosis, Wilson disease, and viral hepatitis was not impressive. Although hepatomegaly, fatty liver infiltration, and steatosis were evident on cross-sectional radiological imaging, the liver biopsy was diagnostic of GH. 

Abdominal ultrasound can detect hepatomegaly; however, it cannot distinguish a fatty liver from GH. An abdominal CT scan provides better visualization of liver densities compared with splenic densities. A relative hyperdensity in the liver favors the diagnosis of GH, and hypodensity favors fatty liver disease [7]. Dual-echo magnetic resonant imaging is a promising imaging modality which detects hepatic deposition of glycogen by identifying low intensities on T2 weighted images and differentiates GH from inflammatory fatty liver disease and NAFLD [8,9]. A recently developed radiological modality, 13C magnetic resonance spectroscopy (MRS), can provide quantitative information of hepatic glycogen deposits; however, it is not available in most medical centers [10]. Liver biopsy is the gold standard for diagnosis of GH, with pale swollen hepatocytes and preserved cellular architecture as the characteristic features on histological examination. In addition, minimal fibrosis, lobular necrosis, and fatty changes may be present. An abundant deposition of cytoplasmic glycogen is the hallmark for GH on periodic acid-Schiff (PAS) staining that disappears with enzymic digestion with diastase staining [2,11].

Tight glycemic control with a stable insulin dosage is the mainstay treatment of GH that results in the normalization of laboratory and histological abnormalities. The patient's counseling for dietary and lifestyle modifications, medication adherence, and close follow-up to ensure good glycemic control, play a vital role in the management of GH and the prevention of recurrent episodes of DKA. The prognosis of GH is excellent, and the majority of patients recover with improved glycemic control. 

## Conclusions

GH is a well-known manifestation of uncontrolled TIDM that could be overlooked due to the underestimation of the disease. The differential diagnosis of RUQ pain, hepatomegaly, and elevated liver enzymes is broad, and a high index of clinical suspicion is required for early diagnosis of GH, especially in patients with poorly controlled TIDM. Although non-invasive modalities such as radiological imaging and elastography are helpful in differentiating it from NAFLD, liver biopsy is essential to rule out other glycogen storage diseases. A close follow-up with patients to ensure medication compliance, adjustment of medications, and counseling about dietary and lifestyle modifications are effective strategies for excellent glycemic control and good prognosis of GH. 
